# Teenage pregnancy matters in refugee setup: early pregnancy among adolescent girls in Kule refugee camp, Gambella, Ethiopia

**DOI:** 10.1186/s12884-023-06178-0

**Published:** 2023-12-14

**Authors:** Girmay Adhena, Arega Fikre

**Affiliations:** 1 Department of Reproductive Health, International Medical Corps, Gambella, Ethiopia; 2 Department of Health, International Medical Corps, Addis Ababa, Ethiopia

**Keywords:** Teenage, Pregnancy, Factors, Refugee, Kule, Ethiopia

## Abstract

**Background:**

An estimated 21 million adolescent girls become pregnant with nearly half of these pregnancies being unintended, and more than half end in unsafe abortion in low and middle-income countries every year. Teenage pregnancy causes serious health, social, and economic consequences around the globe. Despite it is a common problem in the whole community it is more devastating when this occurs in a refugee setup. This study assessed the magnitude of teenage pregnancy in the Kule refugee camp, in Ethiopia.

**Methods:**

A community-based mixed cross-sectional study was done among 422 adolescent girls. Participants were selected using a systematic sampling technique. A structured, pre-tested, and interviewer-administered questionnaire was used to collect the data. Binary and multivariable logistic regression was used to identify associated factors. Adjusted odds ratio with 95% CI was used to show the strength and direction of the association. For the qualitative part, four focused group discussion sessions were done, and participants were selected purposely. Thematic analysis was used to analyze, and the finding was triangulated with quantitative findings.

**Result:**

A total of 146 adolescents (34.6%, 95% CI: (29.9, 38.9)) have experienced pregnancy at least one time. Age (≤ 16) [AOR = 0.48, 95% CI: (0.27, 0.85)], not attending school [AOR = 3.59, 95% CI: (1.2, 10.8)], having a mother with no history of teenage pregnancy [AOR = 0.45, 95% CI: (0.21, 0.98)], being unmarried [AOR = 0.21, 95% CI: (0.12, 0.36)], and having a sister/s with a history of teenage pregnancy [AOR = 2.22, 95% CI: (1.33, 3.7)] were significantly associated factors.

**Conclusion:**

More than one-third of adolescents experience teenage pregnancy. The magnitude of teenage pregnancy was high which may lead to serious health consequences for both the mother and their fetus. Addressing cultural barriers and strengthening adolescent reproductive health education to decrease intergenerational transmission of teenage pregnancy through community awareness and strengthening reproductive parent-adolescent Sexual and Reproductive Health communication are important measures to tackle the problem.

## Background

Teenage pregnancy is defined as a pregnancy in girls between the ages of 13 and 19 years old [1]. The Adolescent age is a phase when enormous physical, psychological, and social changes occur. One in five individuals (around 1.2 billion) in the world is an adolescent [2]. The health and well-being of this age group have important ramifications for the future of nations, regions, and the globe [3].

Teenage pregnancy is a global problem occurring in high, middle, and low-income countries. It is more likely to occur in marginalized communities, commonly driven by poverty and a lack of education and employment opportunities [4]. About 21 million adolescent girls aged 15–19 years old become pregnant and 19 million girls of this age group also give birth in the developing world every year. Near half of these pregnancies were unintended, and more than half ended in unsafe abortion [5].

In low- and middle-income countries, as well as areas of conflict, limited resources, and technical abilities have resulted in a widespread state of neglect concerning adolescent reproductive health [6]. Evidence showed that the pooled prevalence of teenage pregnancy was 18.8% in Africa and 19.3% in Sub-Saharan Africa (SSA) [7]. Teenage mothers accounted for more than half of all births in SSA [8, 9]. Despite the decline of global adolescent birth rates from 65 to 47 births per 1000 women between 1990 and 2015, teenage pregnancies remain unacceptably high especially in sub-Saharan Africa [8, 10].

Despite good progress in reducing the maternal mortality rate (MMR), teenage pregnancy is associated with high maternal mortality and many women are still dying from pregnancy and childbirth-related complications [11]. Moreover, Sustainable Development Goal Three (SDG-3) aims to reduce MMR to less than 70 per 100,000 live births globally by 2030 [12]. Evidence showed that the prevalence of teenage pregnancy in Ethiopia decreased from 16.3% in 2000 to 12.5% [13, 14].

In South Sudan, teenage pregnancy is a major challenge affecting girls and young women’s health and their social, economic, and political empowerment. South Sudan is among the 10 countries with the highest prevalence of child marriage with a prevalence of 52% [4]. According to the National Household and Health Survey, one-third of the 15-19-year-old girls in the country have started childbearing and 96% of women of reproductive ages were not using any contraceptive method [15].

Globally, around 6.6 million adolescents are displaced by war or political conflicts, and a large proportion of this group is living in Africa [16]. Moreover, the statistics show that 90% of the youth in this group experience conflict, poverty, and a lack of opportunity. Within this displaced group, teenage pregnancy is one of the most significant health issues [16, 17].

Early pregnancies have major health consequences for adolescent mothers. It results in a major impact concerning health (preeclampsia, Eclampsia, puerperal endometritis, anemia, abortion, and systemic infections), social (stigma, rejection or violence by partners, parents, and peers, divorce, and drop out of school), and economic consequences [5, 18, 19]. It can also increase the risks for newborns. Babies born to mothers under 20 years of age face higher risks of low birth weight, preterm delivery, stillbirth, prematurity, and severe neonatal conditions [20].

Teenage pregnancy is a major public health problem in Africa, particularly in sub-Saharan Africa. The situation is worse for girls living in refugee, low-resource settings, characterized by poverty, restricted access to care, weak health systems, lack of education and employment, and weak social safety nets [4]. Evidence on adolescent sexual and reproductive health is vital to support decision-making to advance these initiatives and to develop effective programs addressing adolescents’ needs. Yet, numerous data and research gaps impede these efforts mainly in refugee setup. Thus, this study aimed to assess the magnitude of teenage pregnancy and associated factors in the Kule refugee camp, Gambella, Ethiopia.

## Methods

### Study area and period

The study was conducted in the Kule refugee camp, in Gambella, Ethiopia. It is one of the refugee camps which is serving the community of South Sudan in the Gambella regional state. It is located around 45 km away from Gambella town and 821 km away from Addis Ababa, the capital city of Ethiopia. The total estimated population of the Kule camp was 47,201. Out of this number, 25,796 (55%) were females and 21,405 (45%) were males [UNHCR, 2021 report]. The study was conducted from September 21 up to 29/2021. All methods were carried out in accordance with relevant guidelines.

### Study design

Community based mixed cross-sectional study was used.

### Population

All adolescent girls aged 15–18 years old in the Kule refugee camp consisted of the source population. All teenagers aged 15–18 years old from the selected households were the study population. Teenagers who were unable to interview due to severe medical illness were excluded.

### Sample size determination and sampling procedures

For the quantitative part, the sample size was calculated using a single population formula (*n=* (*z a/*2)^2^*p* (1 − *p*))/*d*^2^), where *Z* is the normal standard deviation set at 1.96, with a confidence level of 95% and a tolerable margin of error (*d*) at 5%, 10% non-response rate, and p is the prevalence of teenage pregnancy. Since there was no similar study done in refugee communities of South Sudan that shows the prevalence of teenage pregnancy, the *p*-value was taken as 0.5 (50%). Based on the formula and substituting the values, it resulted in 384, and adding a 10% nonresponse rate (384 + 384*0.1) resulted in 422. The final sample size used for this study was 422. For the qualitative part, the study involved purposely selecting participants in four focused group discussions (FGDs). The FGD participants were selected from the Refugee Committee Council (RCC), women’s affairs, teenagers, religious leaders, and community key figures. Overall, a total of four focused group discussions (12 members for each FGD) were conducted based on their convenience, accessibility, and knowledge of conveying the information.

For the sampling procedure, there were seven zones in the refugee camp with a total of 9417 households. The number of households was considered for each zone and proportion allocation to the sample size was done to identify the required number of households for each zone. The participant was interviewed from the selected household using a systematic sampling technique. The first household was selected using the lottery method, and the next household was identified using a systematic sampling technique every K^th^ value (22). When more than one eligible teenager was found in the selected household, only one respondent was chosen by the lottery method. In cases where eligible was not found in the selected household, then the next household was taken. For the qualitative part, a purposive sampling technique was used to select the focused group discussants. Four focus group discussions (FDG) were conducted with a total of 48 purposely selected participants.

### Data collection tool and procedures

The data were collected using pretested, structured, and interviewer-administered questionnaires. The questionnaire was adapted and developed with modifications from similar previous studies [21–23]. It consisted of socio-demographic, family-related, behavioral, and reproductive health-related items. It was first prepared in English and then translated into the local language (Nuer) by a language expert (person). Data were collected by seven females (degree holders) and intensive supervision was done by two public health professionals (master holders) throughout the data collection period. For the qualitative part, a phenomenology research design was used in this study [24]. A semi-structured, open-ended, and non-directive focus group discussion guide was used and designed to triangulate responses obtained by the structured questionnaire on attitude and perception toward teenage pregnancy [25, 26]. Two master’s holders and two (degree holders) were facilitated FGD. Each group had a facilitator/modulator and recorder/assistant modulator. The facilitator/ moderator interacts with the group, asks questions, and makes notes, and the assistant moderator/recorder handles logistics, arranges the room, welcomes participants as they arrive, takes careful notes, and monitors recording equipment. Data was recorded through radio recordings.

### Operational definition

#### Teenage

A girl whose age was between 15 and 18 years old.

#### Teenage pregnancy

A girl aged 15 to 18 years old who had ever given birth, was pregnant at the time of the survey, or who had ever had a pregnancy terminated [27].

### Data quality assurance

To assure the quality of data, two days of intensive training on data collection tools, ethical issues, and quality of data collection were given to data collectors and supervisors. The questionnaire was translated to the local Nuer language and then retranslated to English by a language expert (person) to check whether the transition is consistent. A pretest was done in 5% (21 individuals) of the sample size in the Terkidi Refugee camp, which is not a part of the study area but the same with socio-cultural characteristics with the sample population. Close follow-up was done by the principal investigators and supervisors during the data collection period.

For the qualitative part, before analyzing the data, all FGDs were transcribed verbatim in the Nuer language and then translated into English by the principal investigator and a research assistant to ensure consistency. The one-day training was given for moderators, tape recorders, and note-takers by principal investigators on the discussion guide, the purpose of the study, and the way to interact with participants. The FGD was conducted in the local language of Nuer which is spoken and well understood by all study participants. Simple language with short well-constructed and understandable questions was used in the discussion. All nonverbal communication such as laughter and disagreements by shaking head was also documented. Questions were asked until there was no new information that was generated from the study participants. At the end of FGD, study participants were given an opportunity to ask questions related to the discussion. The group discussion lasted for 55 to 60 min. A semicircular seating arrangement was used to ensure there was good communication between the study participants and interviewers. Separate note-takers were involved in the process of notetaking. All persons involved in conducting the session of FGD except the moderator refrained from talking. The note-takers produced notes as exact as possible.

### Data analysis

First, data were checked manually for completeness and consistency. Each completed questionnaire was assigned a unique code and entered into Epi-data version 3.1. Then data were exported to SPSS version 20 for analysis. Exploratory analysis was done to find outliers, missed values, and inconsistencies. Frequencies, proportions, and summary statistics were used to describe the study population and presented in tables, figures, and narratives. The bivariate analysis was carried out to identify significantly associated variables with the outcome variable. Co-linearity was checked using VIF and a tolerance test was checked to test model fitness. Those Variables in bivariate analysis whose *p*-value was less than 0.25 were considered candidates for the multivariable analysis. Candidate variables were enrolled in the final model (multivariable analysis) so as not to miss associated factors. Then multiple logistic regression analyses were used to identify independent predictors by controlling the possible confounders. Finally, a variable with a *p*-value less than 0.05 in the final model was considered statistically significant.

For the qualitative part, the data were analyzed using thematic analysis. Information from participants’ descriptions and explanations of their attitudes, opinions, and perceptions towards teenage pregnancy, and reasons for teenage pregnancy was assessed. Raw notes and tape recordings were used to generate transcripts in the local language (Nuer). The principal investigator and research assistant translated and transcribed the notes and recordings and read the transcripts many times to gain a better understanding of the context, and then coding, identification categories, and themes were carried out. The thematic analysis was then triangulated with quantitative findings.

### Ethical consideration

Ethical clearance was obtained from the Haramaya University College of Health and Medical Sciences Institutional Health Research Ethics Review Committee (IHRERC/023/2021). Informed, written and signed consent was taken from parents/guardians, and verbal consent was taken from adolescents for their voluntary participation in the survey. Each participant was clearly informed about the purpose, risk, and benefit of the study. All WHO COVID-19 prevention and control measures (social distancing, sanitizer, and face mask used) were strictly followed throughout the data collection period.

## Result

### Background characteristics of participants and their parents/guardians

A total of 422 adolescent girls were interviewed, making a response rate of 100%. The mean age of participants was 16.72 (SD ± 1.08) years old. More than half (59.7%) of participants were between the age range of 17 and 18 years old. Near to half, 207 (49.1%) of the participants were Protestant in their religion and more than half, 223 (52.8%) of the participants were Gatjiok in their clan. The majority, 364 (86.3%) were attending school and 309 (73.2%) of participants were in primary school at their educational level. More than one-third, 159 (37.7) of the participants were married, out of this, 63 (39.6%) were married before the age of 15 years old, and 59 (37.1%) of them were married by force. Regarding their parents/guardians, more than half, 236 (55.9%) participants reported that they are living with their both biological parents, 322 (76.3%) of their mothers, and 265 (62.8%) of their fathers did not attend formal education (Table 1).

**Table 1:** Background characteristics of participants and their parents/guardians in Kule Refugee camp, Gambella, Ethiopia, September 2021 (*N* = 422)

Characteristics	Category	Frequency	Percentage
Age (Year)	15–16	170	40.3
	17–18	252	59.7
Religion	Protestant	207	49.1
	Catholic	52	12.3
	Ngun-Deng	25	5.9
	7-day Adventist	51	12.1
	Baba Jhon	37	8.8
	Evangelical	30	7.1
	Others*	20	4.7
Clan	Gatjiok	223	52.8
	Lou	94	22.3
	Gajack	72	17.1
	Gawaar	13	3.1
	Bentiew	8	1.9
	Other**	12	2.8
School attendance	In school	364	86.3
	Not in school	58	13.7
Educational level	Cannot read and write.	38	9
	Can read and write. Primary School	12 309	2.8 73.2
	Secondary School	60	14.2
	Collage and above	3	0.8
Occupational level	Student	219	51.9
	Housewife	60	14.2
	Self-employed	82	19.4
	Unemployed	61	14.5
Marital status	Ever Married	159	37.7
	Never Married	263	62.3
Age at first marriage (Year)	≤ 15	63	14.9
	> 15	96	22.7
Characteristics of marriage	Wanted	100	23.7
	Forced	59	13.9
With whom did she live	With both biological Parents	236	55.9
	Either biological parent	76	18.1
	Guardian/adoptive parents	85	20.1
	Alone	9	2.1
	Others***	16	3.8
Educational status of the mother	Cannot read and write.	322	76.3
	Can read and write.	57	13.5
	Primary School	24	5.7
	Secondary School and above	19	4.5
Educational status of the father	Cannot read and write.	265	62.8
	Can read and write.	30	7.3
	Primary School	23	5.5
	Secondary School and above	104	24.6
Marital status of parents	Married	341	80.8
	Divorced	39	9.2
	Widowed	42	10

*Muslim, traditional faith; **= Lake, Bul, Chuluk; ***= Religious leader, Orphan

### Reproductive health, family, and behavioral related characteristics

More than half of the participants, 214 (50.7) have experienced early sexual initiation (≤ 15 years old). About 155 (36.7%) and 75 (17.8%) of participants reported that their sister/s and mother have a history of teenage pregnancy respectively. Regarding parental discussion, 174 (41.2%) discussed family planning, 200 (47.4%) discussed sex-related information, and 158 (37.4%) discussed unintended pregnancy with their parents. About 265 (62.8%) and 315 (74.6%) reported they received family counseling and sex education at school respectively. More than one-fifth (21.6%) of participants ever used any contraceptive method and 50 (11.8%) of the participants knew about emergency contraceptives. Among those who have experienced pregnancy, 127 (86.9%) were pregnant under 16 years old. Out of this number, more than half, 84 (57.5%) reported their pregnancy was unwanted and 83 (56.8%) did not attend antenatal follow-up. About 66 (15.6%) participants drink alcohol and 344 (81.5%) participants reported they have more than five family sizes in their household (Table 2).

Participants shared that forced marriage is highly practiced in the community. The culture of the community plays a role in the acceptance of early marriage and there is also male dominancy. There is also a restriction on movement anywhere unless they did not get permission from the partner.

*“When a girl becomes 15 and above, she should be married. Because it is a time for her to have a husband and family. Unless in our culture we consider she is spending her time. In addition to this, her family may not keep her until she becomes 18 even, they will forcibly marry her [56 years old man, FGD*_*4*_*]”*.

*“In our community, young girls are married by force and their husbands, or their families do not allow them to go anywhere except they did not get permission from their husbands. They did not go to the health facility, and they do not know about family planning use. So, they become pregnant and face a very difficult situation. I was married before I was 14 years old, and I suffered a lot. I do not want to see in my child the problem that I faced when I was a young girl [48 years old woman, FGD*_*1*_*]”*.

Participants shared that the refugee women mainly adolescents’ girls move long distance from the camp to the other area either for work or asking family on foot. During this time the women will have high chance of rape and risk for unintended pregnancy and other health consequences.

*“There are many reasons that young ladies become pregnant in our community. Among those rape is one of the major reasons. As we refugees move from one place to another which leads to being raped and violated and you do not have an option on how to protect yourself from this serious issue. After the young lady has been raped, she has no choice except to marring that guy who raped her [28 years old woman, FGD*_*4*_*]”*.

Participants described that the role of parents and neighbors play an important issue. Adolescents learn more from their families and neighbors. In addition to that the adolescents learn and follow their sister’s experience.

*“In our family, my sisters gave birth early and we have a big family. When I observe my sisters’ children, I feel very happy, and I always tell them to have a big family like them. My mom also gave birth when she was 15 years old. In our community, if you have children, they will consider you rich. By following my sisters, I also gave birth when I was 16 years old and now, I have 2 children. In my first pregnancy, it was a difficult time for me in my life [18 years old girl, FGD*_*3*_*]”*.

*“There are young girls who are pregnant and gave birth in my neighborhood. Even though I was pregnant and gave birth when I was 14 years old. During the pregnancy and delivery time, ……. I suffered a lot because everything was new to me and very difficult. Especially, during delivery, I was delivered at home and ………hhhh…. Beje ……Beje (feel painful). Even though I tried to advise my child not to get what I got she refused my advice, and she is pregnant now at 16 years old [35 years old women FGD*_*1*_*]”*.

Participants described that many adolescent girls become pregnant and drop out of school. Even though there is an improvement but still, family and partners still do not allow them to go to school. They only restrict them to caring about family and household issues.

*“When I was young that time was difficult, during that time there was no way to know your status and no school. My sisters and brothers did not attend school, rather they took care of their family. But today it is good because girls get the chance to spend their time searching for knowledge and jobs which help them in their future life. Especially girls need to learn more to decrease the cultural challenges that are practiced in our community [a 64-year-old man, FGD*_*4]*_.

*“I am a 7-month pregnant. I was attending a class before I married but my parents instructed me to marry by force a guy that I did not know. I was highly interested in attending my school, but it is very difficult for me to attend school and care for my family at this time. Even though I am interested to learn, I dropped out of school, and I am giving care to my family [18 years old girl FGD*_*2*_*]”*.

**Table 2:** Reproductive Health, Family, and behavioral-related characteristics of participants in Kule refugee camp, Gambella, Ethiopia, September 2021 (*N* = 422)

Characteristics	Categories	Frequency	Percentage
Mother’s history of teenage pregnancy (≤ 18)	Yes	75	17.8
	No	83	19.7
	I do not know	264	62.6
Sister/s history of teenage pregnancy (≤ 18)	Yes	155	36.7
	No	267	63.3
Age at first sex (year)	≤ 15	214	50.7
	> 15	103	24.4
Family size	≤ 5	78	18.5
	> 5	344	81.5
Discussion with a parent on family planning	Yes	174	41.2
	No	248	58.8
Discussion with a parent on sex education	Yes	200	47.4
	No	222	52.6
Discussion with a parent on unwanted pregnancy	Yes	158	37.4
	No	264	62.6
Received family counseling	Yes	265	62.8
	No	157	37.2
Contraceptive use	Ever used.	91	21.6
	Never used	331	78.4
Received sex education at school	Yes	315	74.6
	No	107	25.4
Knew the accurate time to take emergency contraceptive	Know	50	11.8
	Did not Know	372	88.2
Access to social media	Yes	207	49.1
	No	215	50.9
Drink Alcohol (Alcohol Use)	Yes	66	15.6
	No	356	84.4
Number of pregnancies	One time	96	22.7
	Two times	45	10.7
	≥Three times	5	1.2
Age at first pregnancy (Year)	15–16	127	30
	17–18	19	4.5
Characteristics of the pregnancy	Planned	62	14.7
	Unplanned	84	19.9
Antenatal follow up	Attend	63	14.9
	Did not attend	83	19.7

### Magnitude of teenage pregnancy

**Fig. 1:**
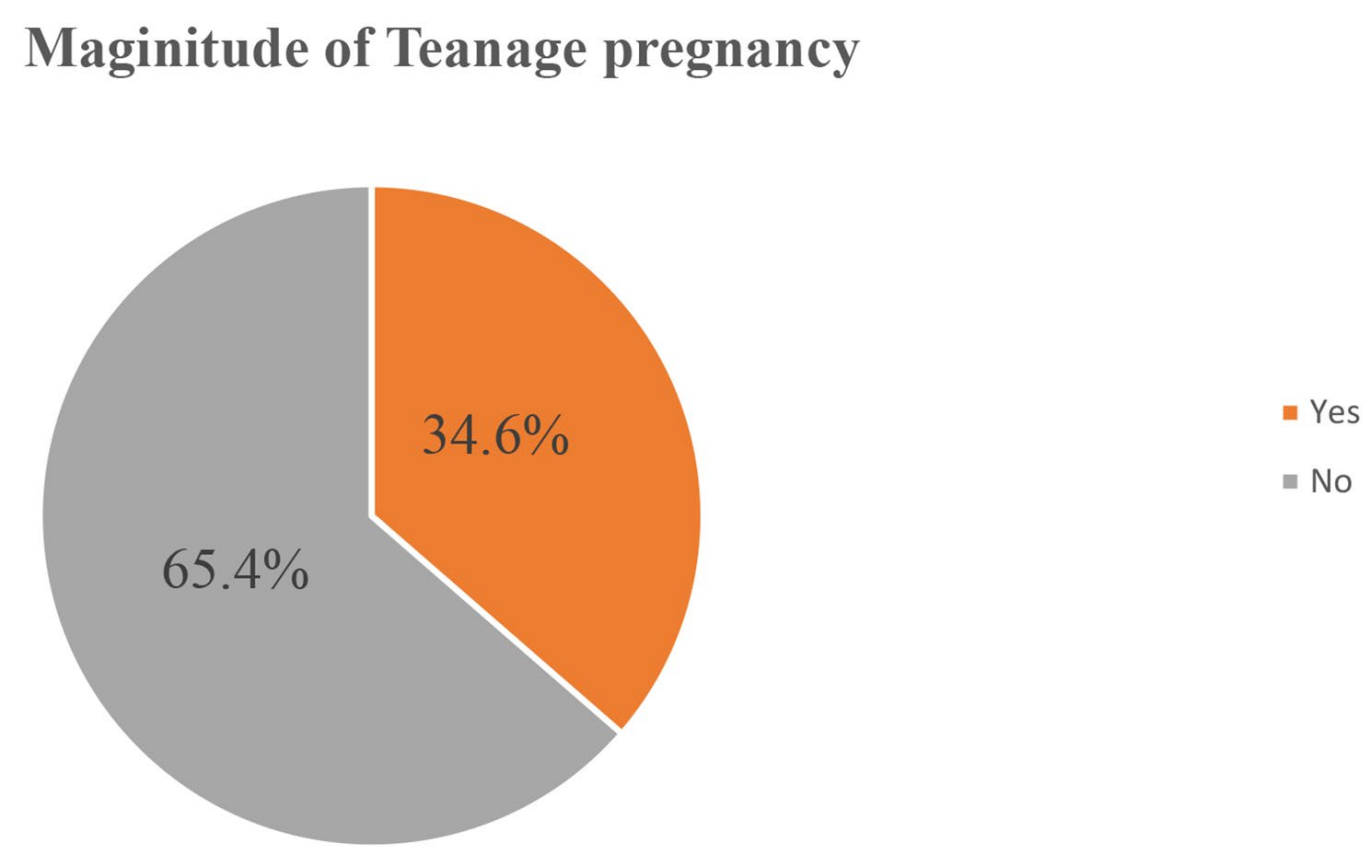
Magnitude of teenage pregnancy in Kule refugee camp, Gambella, Ethiopia, 2021 (*N* = 422)

Among the total of 422 interviewed participants, 146 (34.6%, 95% CI: (29.9, 38.9)) of them have experienced pregnancy at least one time (Fig. 1).

Participants discussed the causes of early marriage of adolescent girls in the community. They considered it as a means of income. When a girl gets married there is a payment that the husband should pay to her family. The dowry that is paid for marriage is the cause that adolescent girls are forced to marriage.

*“In Kule, there are a lot of young girls who are pregnant. When I observed them, I felt very sad because they do not have any access to food and health care. Many causes lead to an increase in the number of adolescent pregnancies in our community. Among those reasons, one is the shortage of property. Adolescents are posing pregnancy in this refugee. When we were in South Sudan, this case was less compared to this area. We lost our family and our property in South Sudan due to the war. Many adolescents including me accepted early marriage because the person that we married would pay a lot of property to our family. To gain these properties that support to our family by the husband by either money, caws and/or whatever our family requested him that is why we married early and become pregnant [18 years old girl, FGD*_*3*_*]”*.

*“When you have five and above daughters, you are rich because when they marry you will get dowery. The husband of your daughters will give you a lot of cows to take his wife. So, girls should respect their cultures and, they should respect their family [a 51-year-old man FGD*_*3*_*]”*.

Participants shared that the culture of the community have a negative impact on the health of the adolescent and the child. The community encourages early marriage and to have many families. If the adolescent girl refuses the interest of her family, they punish and neglect her from her family.

*“Oh…Jege (not good) ….in our culture the community highly encourages early marriage and having many children. In addition to this, the family restricts you from seeing your child because you are young. The young ladies did not properly care for their children because they are too young, and they do not have knowledge of how to care for the child. They did not give proper nutrition such as breastfeeding because the adolescents spend their time playing with their friends rather than caring for their child because they are too young [17 years old girl, FGD*_*3*_*]”*.

*“When girls get married, they do not have any chance except to accept the instruction of the husband and their/his family. If a girl does not marry, she will be neglected by the community and her family especially when she stays until 17 or 18. Some young ladies became pregnant, and their families refused and punished them in my neighborhood and my family. Having a child while you did not marry is risky for violence in our culture [a 49-year-old male, FGD*_*3*_*]”*.

### Parent-adolescent communication on reproductive health issues

**Fig. 2:**
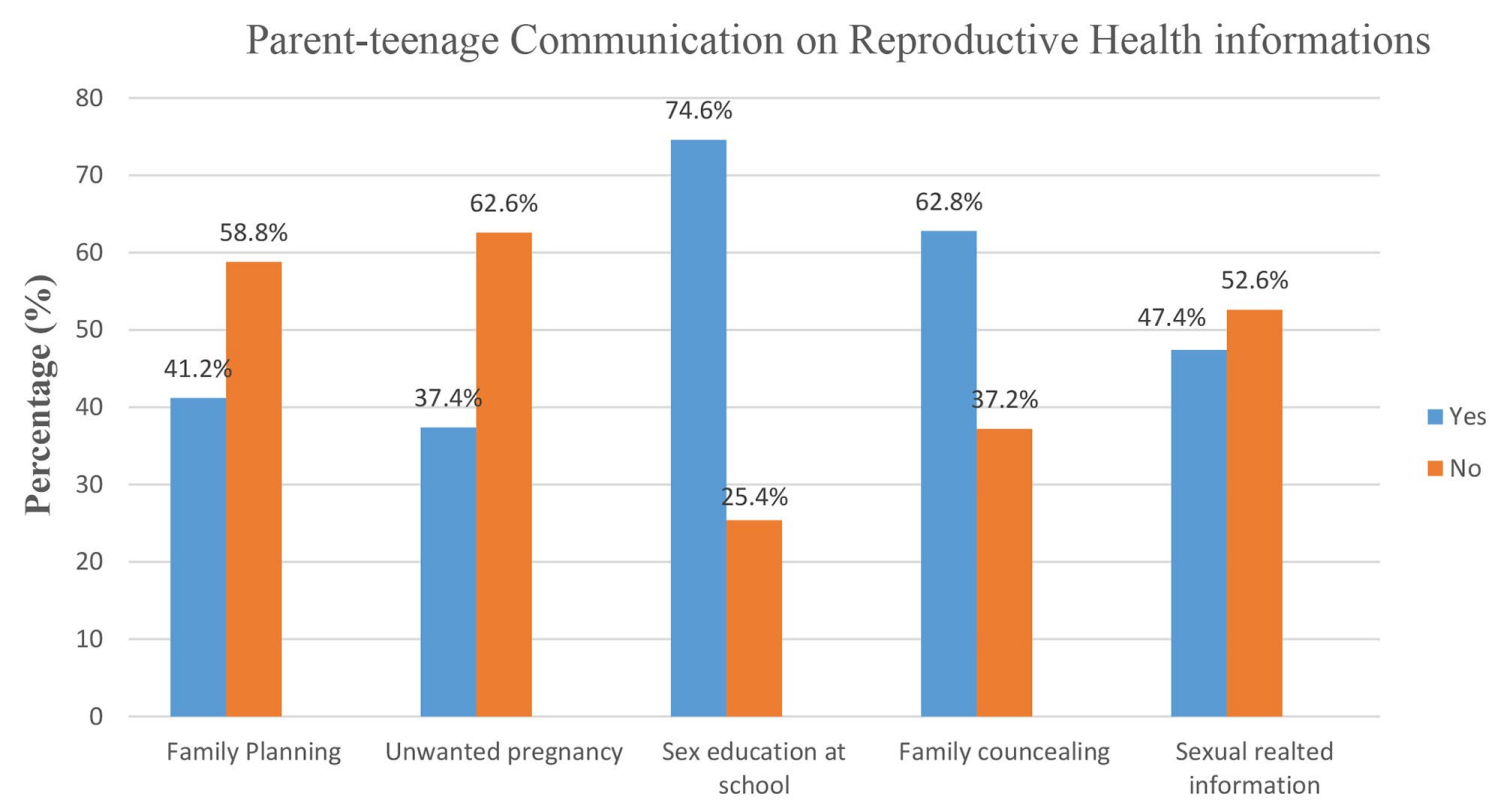
Parent-adolescent communication on reproductive health information in Kule refugee camp, Gambella, Ethiopia, 2021 (*N* = 422)

Regarding reproductive health-related information, three-fourths (74.6%) of participants reported they received sex education at school. About 42% discussed family planning with their parent, 37.4% discussed unintended pregnancy, 62.8% received family counseling, near to half (47.4%) received sex-related information from their parents (Fig. 2).

Parents role on the discussion and awareness plays an important impact on the future life of adolescent girls. Despite their positive role they also have pressure on early and forced marriage.

*“Mothers and other family members have a role in advising their children. But in some ways, the mother and the family member have pressure to practice early marriage and to be pregnant. So, all family members should play a role in preventing early marriage. We should not focus only on adolescent girls the issue also needs to be addressed to adolescent boys [45 years old woman, FGD*_*1*_*]”*.

### Factors associated with teenage pregnancy

In the Binary logistic Regression, age, school attendance, educational status, occupational status, marital status, with whom did she live, educational status (mother), mother history of teenage pregnancy, educational status (father), siter/s history of teenage pregnancy, contraceptive use status, knowledge of emergency contraceptive, access to media and drinking status were significantly associated factors.

In the final model (multivariable regression), age, marital status, maternal/sisters’ history of teenage pregnancy and school attendance were significantly associated factors. Adolescents aged below 16 years old were 52% less likely to experience pregnancy (AOR:0.48 (0.27, 0.85)), those who were not attending school were 3.59 times more likely to experience pregnancy (AOR: 3.59 (1.2, 10.8)), teenagers whose mothers have no history of teenage pregnancy were 55% less likely to experience pregnancy (AOR = 0.45 (0.21, 0.98)), those teenagers who did not married were 71% less likely to experience pregnancy (AOR = 0.21 (0.12, 0.36)), and those whose sister/s had a history of teenage pregnancy were 2.2 times more likely to experience (AOR = 2.22 (1.33, 3.7)) compared to those whose sisters have no history of teenage pregnancy (Table 3).

**Table 3:** Factors independently associated with teenage pregnancy among adolescent girls in Kule Refugee Camp, Gambella, Ethiopia, September 2021 (*N* = 422)

Characteristics	Teenage pregnancy		COR (95% CI)	AOR (95% CI)
	Yes (%)	No (%)		
Age (Year)				
15–16	31 (18.2)	139 (81.8)	0.266 (0.17, 0.42)	0.48 (0.27, 0.85) *
17–18	115 (45.6)	137 (54.4)	Ref.	Ref.
School attendance (currently)				
In school	114 (31.3)	250 (68.7)	Ref.	Ref.
Not in school	32 (55.2)	26 (44.8)	2.96 (1.54, 4.74)	3.59 (1.2, 10.8) *
Educational status				
Not attended formal education	23 (45.1)	28 (54.9)	0.96 (0.41, 2.12)	0.21 (0.06, 1.02)
Primary School	96 (31.2)	212 (68.8)	0.53 (0.305, 0.92)	1.2 (0.6, 2.4)
Secondary School and above	29 (46)	34 (54)	Ref.	Ref.
Occupational status				
Student	61 (27.9)	158 (72.1)	7.1 (3.74, 13.56)	2.69 (1.07, 6.82)
Housewife	44 (73.3)	16 (26.7)	1.07 (0.61, 1.88)	1.08 (0.56, 2.09)
Self-employed	24 (29.3)	58 (70.7)	1.00 (0.53, 1.88)	1.2 (0.57, 2.5)
Unemployed	17 (27.8)	44 (72.2)	Ref.	Ref.
Marital status				
Married	97 (61)	62 (39)	Ref.	Ref.
Never Married	49 (18.6)	214 (81.4)	0.15 (0.09, 0.23)	0.21 (0.12, 0.36) **
With whom did she live?				
With both biological Parents	85 (36)	151 (64)	Ref.	Ref.
Either biological parent	20 (26.3)	56 (73.7)	0.63 (0.36, 1.13)	0.46 (0.22, 0.95)
Guardian/adoptive parents	34 (40)	51 (60)	1.18 (0.71, 1.97)	0.74 (0.39, 1.4)
Others	7 (28)	18 (72)	0.69 (0.28, 1.72)	0.44 (0.14, 1.35)
Educational level mother				
Cannot read and write.	123 (38.2)	199 (61.8)	1.59 (0.47, 3.2)	1.63 (0.61, 5.4)
Can read and write.	11 (19.3)	46 (80.7)	0.58 (0.21, 1.32)	0.45 (0.4, 1.53)
Primary School and above	12 (27.9)	31 (72.1)	Ref.	Ref.
Educational level (father)				
Can not read and write.	104 (39.2)	161 (60.8)	1.92 (1.16, 3.21)	1.47 (0.74, 2.8)
Can read and write.	10 (33.3)	20 (66.7)	1.48 (0.61, 3.59)	1.76 (0.52, 5.96)
Primary School and above	32 (25.2)	95 (74.8)	Ref.	Ref.
Mother’s history of teenage pregnancy				
Yes	38 (50.7)	37 (49.3)	Ref.	Ref.
No	27 (32.5)	56 (67.5)	0.47 (0.25, 0.89)	0.45 (0.21, 0.98) *
Do not know	81 (30.7)	183 (69.3)	0.43 (0.26, 0.72)	0.36 (0.17, 1.02)
Contraceptive use				
Ever use.	38 (41.8)	53 (58.2)	Ref.	Ref.
Never use	108 (32.6)	223 (67.4)	0.68 (0.42, 108)	0.87 (0.44, 1.73)
Sister/s history of teenage pregnancy				
Yes	70 (45.2)	85 (44.8)	2.07 (1.37, 3.13)	2.22 (1.33, 3.7) **
No	76 (28.5)	191 (71.5)	Ref.	Ref.
Knowledge of time of emergency FP				
Good	24 (48)	26 (52)	Ref.	Ref.
Poor	122 (32.8)	250 (67.2)	0.53 (0.29, 0.96)	0.62 (0.29, 1.29)
Access to media				
Yes	78 (37.7)	129 (62.3)	Ref.	Ref.
No	68 (31.6)	147 (68.4)	0.76 (0.51, 1.14)	0.61 (0.37, 1.01)
Drinking status (alcohol)				
Used	30 (45.4)	36 (54.6)	1.72 (1.01, 2.94)	1.58 (0.75, 3.33)
Did not use	116 (32.6)	240 (67.4)	Ref.	Ref.

∗ = *p*-value < 0.05; ∗∗ = *p*-value < 0.01; CI = confidence interval; COR = crude odds ratio; AOR = adjusted odds ratio

## Discussion

Overall, the magnitude of teenage pregnancy in the Kule refugee camp was 34.6% (95% CI: (29.9, 38.9)). Age, school attendance, mother’s history of teenage pregnancy, marital status, and sister/s history of teenage pregnancy were significantly associated factors with teenage pregnancy.

The magnitude of teenage pregnancy in this study is in line with the findings from Benin (30.2%) and Eastern Ethiopia (30%) [22, 28]. However, it is lower than from studies done in Cameron (60.75%), Malawi (76%), Ethiopia (79.6%), and the Demographic Health Survey (DHS) in Sub-Saharan African countries (44.3%) [27, 29–31]. The possible reason for the discrepancy might be due to the difference in study design, sample size, the age of study subjects, and duration of the study. For example, a study in Ethiopia was done on participants aged 20–24. The duration of the study done in Sub Sharan Africa was from 2010 to 2018 which varies duration, the study in Cameron was also a clinic-based study in which pregnant were easily accessed antenatal care whereas this study is a community-based study this might be the reason for the discrepancy.

The finding of this study is higher than studies done in Canada (4.3%), DHS in East Africa (29%), DHS in Nepal (17.3%), South Africa (19.9%), Wegadi (Ethiopia) (28.6%), DHS in Ethiopia (10%), Arba Minch (7.7%) [21, 23, 32–36]. The reason for the difference might be due to the difference in the study setting, study subjects, and sample size. For example, in a study in Canada and South Africa, the educational and economic status of these study subjects was different from the refugees. They may have a good education and access to health care whereas in this study participants are refugees who may not access the health and school services compared to South Africa and Canada. The discrepancy might be also due to the study design, example the study in Arba Minch was done in the school whereas this study is a community-based study which may give the chance to access easily to those pregnant who did not attend school and drop out of school due to the pregnancy.

This study tried to assess factors associated with teenage pregnancy. Adolescent girls aged 16 and below were 52% less likely to experience pregnancy compared to those aged 17 years old and above. This is consistent with the study done in Ethiopia, Wegedi, Eastern Ethiopia, and DHS in Sub-Saharan Africa [21, 22, 27, 37]. A possible reason for this might be those adolescents aged 17 and above are minor mature and they may increase their interest in having a child compared to those below 16 years old. In addition to this, the culture of the community may also be a reason to increase the experience of teenage pregnancy.

Teenagers who did not marry were 79% less likely to experience pregnancy compared to married teenagers. This is supported by the findings done in Eastern Ethiopia [22]. The possible reason for this might be that those who did not marry may have less risk of practicing sexual intercourse and those married may increase their interest in having a child in addition to the influence of family and partner. In addition to this, the cultural value of the community to marriage may also affect the experience of teenage pregnancy. The other reason might also be that those who are married may not use contraceptives due to the refusal of their partner which may increase the occurrence of pregnancy.

Teenagers whose mothers have no history of teenage pregnancy were 55% less likely to experience pregnancy compared to those who reported their mothers had a history of teenage pregnancy. This is consistent with the finding from Degua Temben (Ethiopia) [38]. Teenagers who reported that their sister/s had a history of teenage pregnancy were 2.2 times more likely to experience pregnancy compared to those whose sister/s had no history of teenage pregnancy. This is consistent with the study done in Eastern Ethiopia [22]. A possible reason might be that adolescents might share information and experience with their family members. The other possible reason may also be the pressure of their family to marry and have a child.

In this study, adolescent girls who were not attending school were 3.59 times more likely to experience pregnancy compared to those who were attending school. This finding is supported by the finding from Eastern Ethiopia [22]. The reason for this may be that those who did not attend school might drop out of school due to family pressure and they may not receive information about the risk of early marriage which may increase the occurrence of pregnancy. Those who attend school may give time to their education and may also have a chance to get information about the consequences of teenage pregnancy in school.

## Conclusion

Overall, more than one-third of adolescents experienced pregnancy at least once. The magnitude was high which may lead to serious health consequences for both the mother and her fetus. Being in the age category of 15–16, not attending school, having a mother and sister/s with a history of teenage pregnancy, and marital status were significantly associated factors. Addressing cultural barriers and strengthening adolescent education to decrease intergenerational transmission of teenage pregnancy and early marriage through community awareness and parental involvement are important measures to tackle the problem. The longitudinal study is also recommended for researchers to further investigate the problem.

## Data Availability

The datasets used and/or analyzed during the current study are available from the corresponding author (Girmay Adhena) upon reasonable request.

## Abbreviations

DHS: Demographic and health survey
FGD: Focused group discussion
MMR: Maternal mortality rate
RCC: Refugee Central Committee
SSA: Sub-Saharan Africa
UNHCR: United nation Higher Commission for Refugees
WHO: World Health Organization
